# The feasibility of implementing risk stratification into a national breast cancer screening programme: a focus group study investigating the perspectives of healthcare personnel responsible for delivery

**DOI:** 10.1186/s12905-022-01730-0

**Published:** 2022-05-02

**Authors:** David P. French, Victoria G. Woof, Helen Ruane, D. Gareth Evans, Fiona Ulph, Louise S. Donnelly

**Affiliations:** 1 Division of Psychology & Mental Health, Manchester Centre for Health Psychology, School of Health Sciences, University of Manchester, Manchester, UK; 2 Nightingale & Prevent Breast Cancer Research Unit, Manchester University NHS Foundation Trust, Manchester, UK; 3 Division of Evolution and Genomic Science, Department of Genomic Medicine, University of Manchester, Manchester, UK; 4 Division of Population Health, Health Services Research & Primary Care, School of Health Sciences, University of Manchester, Manchester, UK

**Keywords:** Breast screening, Healthcare professionals, Implementation, Risk stratification, Focus groups, Thematic analysis

## Abstract

**Background:**

Providing women with personalized estimates of their risk of developing breast cancer, as part of routine breast cancer screening programmes, allows women at higher risk to be offered more frequent screening or drugs to reduce risk. For this to be feasible, the concept and practicalities have to be acceptable to the healthcare professionals who would put it in to practice. The present research investigated the acceptability to healthcare professionals who were responsible for the implementation of this new approach to screening in the ongoing BC-Predict study.

**Methods:**

Four focus groups were conducted with 29 healthcare professionals from a variety of professional backgrounds working within three breast screening services in north-west England. An inductive-manifest thematic analysis was conducted.

**Results:**

Overall, healthcare professionals viewed the implementation of personalised breast cancer risk estimation as a positive step, but discussion focused on concerns. Three major themes are presented. (1) *Service constraints* highlights the limited capacity within current breast services and concerns about the impact of additional workload. (2) *Risk communication* concerns the optimal way to convey risk to women within resource constraints. (3) *Accentuating inequity* discusses how risk stratification could decrease screening uptake for underserved groups.

**Conclusions:**

Staff who implemented risk stratification considered it a positive addition to routine screening. They considered it essential to consider improving capacity and demands on healthcare professional time. They highlighted the need for skilled communication of risks and new pathways of care to ensure that stratification could be implemented in financially and time constrained settings without impacting negatively on women.

**Supplementary Information:**

The online version contains supplementary material available at 10.1186/s12905-022-01730-0.

## Background

Although breast cancer incidence continues to rise internationally, deaths have fallen over the last 25 years. This is thought to be, in part, due to the introduction of routine mammographic screening leading to early detection of cancers at a more treatable stage [1, 2].

However, there continues to be controversy over the balance of benefits of breast cancer screening to harms such as over-detection and false positive screening test results [1]. To improve this balance, there is increasing interest internationally in risk-stratified screening [3]. Risk-stratified screening involves estimating each woman’s risk of breast cancer, and further prevention or treatment options being available depending on that woman’s risk. For instance, it has been recommended since 2013 that women in the UK at high-risk of breast cancer should have more frequent screening between ages 40–60 years and be offered risk-reduction strategies, such as chemopreventive therapy [4]. A major barrier to the implementation of these recommendations is that only a minority of women are aware of their breast cancer risk [5].

A number of trials are underway to evaluate the potential harms and benefits of risk-stratified screening [3]. One such trial is the Breast Cancer Predict (BC-Predict) study being conducted in three UK breast screening services in the North-West of England [6]. In the BC-Predict study, women who are in the risk-stratified screening condition have been invited to complete a risk questionnaire ahead of their scheduled screening mammogram, which was combined with an assessment of the woman’s breast density to give an personalised estimation of developing breast cancer in the next 10 years The risk estimates were provided as categories with numerical and text labels, i.e. “high” (8% risk or higher), “moderate” (5 to 7.99%), “average” (2 to 4.99%) or “low” (less than 2%), following patient involvement recommendations [5]. There is considerable evidence of the validity of such breast cancer risk estimates from a previous study of 58,000 women [7]. Risk feedback is being provided by letter approximately 6–8 weeks after the screening mammogram.

A focus of the BC-Predict study is on the feasibility of carrying out risk stratification, including the provision of appropriate follow-up care pathways involving risk consultation, chemoprevention for moderate/high risk women (≥ 5% 10 year risk), and more frequent mammography for moderate risk women aged < 50 years and high-risk women aged up to 60 years. The integration of complex pathways into existing screening services is a source of concern for healthcare professionals [8]. However, previous studies with healthcare professionals to date have asked them about the implementation of risk stratification in the abstract. The present study asked healthcare staff for their views on developing and delivering optimal pathways as part of formative work for BC-Predict, with the expectation that these pathways were to be implemented for 8 months at the sites where they worked as part of this research study. The present study research was undertaken prior to the BC-Predict trial, to inform the development of research procedures and care pathways in that trial.

The objective of the current qualitative study was to investigate National Health Service (NHS) staff perspectives on the optimal pathway for delivering personalised breast cancer risk feedback for all women, and further prevention and early detection services within the NHS Breast Screening Programme (NHS-BSP) and Family History Risk and Prevention Clinics for women identified as being at higher risk of breast cancer.

## Methods/design

Methods are reported in accordance with the Consolidated criteria for Reporting Qualitative Research (COREQ) [9], with the checklist provided as Additional file 1.

### Study design and participants

Four focus groups were conducted with 29 NHS personnel, across three breast screening services in the North West of England (Table 1). The three sites were chosen on the basis that the pathways developed and refined by the present research were to be implemented for 8 months at the sites where they worked. The research was therefore part of a larger research study run by the same research team [6], and had involved ongoing discussions over a period of years between members of the research team and some of the research participants. Participants were recruited by liaising with practice managers and heads of service to identify key members of their service. Potential participants were then sent the participant information sheet via email or post and asked to contact the research team if they were interested in participating in a one-off focus group to discuss the prototype care pathway for implementing risk estimation and stratification into the breast screening programme. Thirty individuals who were invited to the study did not participate, with limited capacity most typically given as the reason.

**Table 1:** Focus group participants

Occupation	Participants
Radiographer/mammographer	9/1
Consultant Radiologist	5
Clinical Fellow (Radiology)	2
Breast Screening Office Manager	2
Advanced Nurse Practitioner	2
Family History Risk & Prevention Clinic Nurse	1
Clinical Nurse Specialist (Breast)	6
General Practitioner	2

Focus groups were considered the most effective method of data collection in order to identify the most important modifications to the current breast screening pathway and to prioritise contradictory suggestions of the diverse group of professionals involved at differing points along the breast screening pathway. Focus groups were conducted at the respective breast screening centres and lasted between 90 and 120 min. Each group was preceded by a presentation by one member of the research team (DPF, DGE or FU) who described the overall study and the proposed initial pathways, answered any outstanding questions from participants and then left before the focus group started. The groups were facilitated by two female qualitative researchers; LD (research fellow) and HR (research assistant) who clearly described their role as wanting to make the proposed pathways workable for research participants. The lead researcher (LD) had a PhD focussing on qualitative methods, and had over 10 years of post-doctoral experience as a qualitative researcher; the other researcher (HR) had recently attended a training course in qualitative methods run by University College London and had worked on several qualitative research projects. The focus groups were guided by a schedule developed by the wider team, and field notes made during the focus groups.

### Analysis

Data were audio recorded, transcribed, then analysed in NVivo version 11 using Thematic Analysis [10]. Thematic analysis enables the identification of representative patterns of participants’ views and experiences from across the data set. Data were analysed from a realist perspective, viewing the data as representing the truth of experience and resulting in a rich and data driven analysis. Each transcript was systematically read multiple times for familiarisation prior to coding by LD and VW (coding tree included as Additional file 2). Coding was carried out at an inductive-manifest level allowing for participants’ subjective views and experiences to be represented, with the view of this analysis informing healthcare practice. Coding was iterative with developing codes compared and refined across transcripts. Patterns were identified within the codes and initial themes were created by LD, HR and VW. Themes and codes were compared across the data set and saturation was achieved when no new codes were generated from the data. Codes, developing themes and the final thematic structure was reviewed and refined by LD, VW, FU, DF, and HR. Transcripts were sent back to participants due to the participants having very limited time for this research. We did not think it appropriate to member check the results, with earlier advocates later acknowledging that this process was philosophically flawed [11].

## Results

Three major themes relating to implementing risk stratification into the NHS-BSP are presented. Quotations are provided to illustrate each theme and sub-theme, and are identified by their professional role.

### Theme 1: service constraints

Participants described how they currently experience system constraints including; a lack of resources, workforce, and time to provide an effective service, making implementing risk stratification into the current NHS-BSP potentially problematic.

#### Demands on health care professional time

Radiographers raised concerns regarding their capacity to answer questions about risk assessment at screening when appointments are time limited (reportedly 7 min). Participants identified that radiographers may be the first HCPs women come into contact with after receiving initial information about breast cancer risk stratification, so may come seeking answers about completing the risk assessment:

*… when the woman attends, it's likely that the person that's dealing with them later, is going to be, get all the questions, and they won't have the answers (Radiographer 5 – site B)*

The Clinical Nurse Specialists and GPs argued that telephone queries and personalised risk consultations will require considerable time and resources due to questions women will have about their health, family history, and care on receiving their risk estimate:

*These ladies are coming for their screening mammogram, then being asked about their family history which they might not have thought about before, or even considered that they might be at risk, so that’s going to increase our workload (Clinical Nurse Specialist 1 – site B)*

The Clinical Nurse Specialists across all sites felt that additional work would disproportionally fall to family history nurses, who have limited staffing and capacity.

#### Impact on overstretched family history risk and prevention clinics

Participants stated that family history risk and prevention clinics are currently overstretched, with some clinics reduced to one morning a month meaning capacity for additional risk consultations would be shared between those identified at increased risk via the NHS-BSP and those referred for risk consultation from General Practice. Nurses expressed concern as to how these additional women would be accommodated:

*We have one [Family History Clinic] a month, we can have 10 patients and often we don’t even have a room to do that clinic in (Clinical Nurse Specialist 1 – site B)*

In addition, women having completed risk assessment prior to attending a consultation was considered problematic as rectifying questionnaire errors would impact on the length and complexity of the consultation.

*…when we actually go through it with them they say “oh yeah, there’s this other person. I know I didn’t put this on. And actually that wasn’t quite right”. So it might end up being a full blown consultation… (Clinical Nurse Specialist 4– site C)*

#### Insufficient resources

Participants across all sites stated that sufficient funding would need to be made available to support risk stratification. Both radiology and nurse participants acknowledged that identifying and hiring skilled personnel to fulfil instrumental roles to deliver an effective service is already challenging without the added pressure of risk stratified screening.

*…how is [Minister for Health] gonna get the money together, then, to fund all this? Because it's difficult, there's a difficulty recruiting the clinical team, that's the radiologist, the doctors, the radiographers, all that, very, very difficult (Radiographer 5 – site B).*

*…you can throw money at something but it doesn’t make a difference. You need people with the appropriate skills to be doing the job (Consultant Radiologist 5– site C)*

Participants questioned whether a risk adapted screening programme could be run effectively and, if not, what sort of impact an insufficient service would have on women’s wellbeing:

*…the last thing you want then is to identify a woman and you're struggling with the resources for the next step…And then the woman's having to wait, and that will increase her anxiety (Radiographer 5 – site B).*

### Theme 2—risk communication

Strong views were expressed regarding the presentation and method of receiving risk. Concerns centred on dissemination of risk, not that risk should not be conveyed.

#### Risk letter content

Current intentions are to communicate risk via letter. The clarity of those letters was a significant consideration for the Radiologists and Clinical Nurse Specialists. It was suggested that contextual information, drawing women’s attention to the implications of their risk, and facilitating their understanding of how they compare to others should be presented clearly and explicitly:

*A very well worded letter that was in layman's terms, that was giving a risk equivalent to other things...Where they could understand by reading it what the implications potentially would be (Consultant Radiologist 5 – site C)*

It was also suggested that women should be given a visual representation of their risk to mitigate any confusion in interpreting percentages:

*So you’ve got a hundred smiley faces, and then you’ve got two [sad faces]. And so I think sometimes, visually, it looks so much better, than saying, five to seven per cent, and you think, oh no, that’s me (GP2 – site B)*

Furthermore, it was suggested that risk letters containing complex information may not be accessible for some individuals, such as those with low literacy skills. A suggestion was made to create a letter which clearly provides women with optional reading material:

*…would it be possible to create a letter that, for example, states the basic principles of what we want to tell them and ask them on one page only and then for people who want to read more into it, a second part that is more detailed (Clinical Fellow 1– site A)*

#### Impact of risk letters on well-being

Participants at site B expressed uncertainty as to whether communicating high-risk results through a letter is acceptable due to the likelihood of it causing anxiety and distress, *‘…if I got a letter to say I was at high-risk, I would be freaked, I would’ (Radiographer 6 – site B).* Further to this, concerns were raised with regards the appropriateness of sending moderate and high-risk women their results by letter:

*I really have reservations over telling someone they’re high-risk in a letter…I personally would never do that (Consultant Radiologist 3- site B)*

Despite concern, GPs stated that risk in other diseases is communicated via letter and the possibility of causing anxiety is inevitable in spite of how carefully results are disseminated:

*…it does sound quite scary being told that your breast cancer risk is whatever. But we do that a lot for heart disease, and I think patients are used to it now (General Practitioner 2 – site B)*

#### Communication of high-risk referral process

Participants unanimously agreed that high-risk women should receive a face-to-face consultation about their care options upon receiving risk results, ‘*The high-risk ones, they're going to come to a clinic, aren't they? Because I think that's appropriate’ (Consultant Radiologist 5 – site C).* It was suggested that these women should receive an automatic appointment, ‘*…if they are known to have a high-risk, they could automatically get an invitation to the family history clinic’ (Radiographer 1 – site A).* The HCPs recommend that a telephone hotline should be accessible and this information should be made explicit in the letters in order for women to gain immediate answers whilst waiting for risk consultations:

*I do think that they need a point of contact. Anybody who gets that result, they panic, they think “oh god what does it mean” and so they will need somebody to talk to (Advanced Nurse Practitioner 1 – site B)*

However, some of the Clinical Nurse Specialists expressed a preference to encourage women to have telephone consultations due to limited capacity at family history risk and prevention clinics.

### Theme 3—accentuating inequity

Pre-existing issues surround both uptake to screening and pathways/guidelines for care, which the addition of risk provision could accentuate.

#### Women’s personal barriers

Participants stated that disengagement in breast screening is a continual problem. They attributed this to women’s personal health anxieties, stress, and reservations about breast screening. Screening staff at all sites were concerned with the balance of meeting screening targets and suggested risk stratification could be an excuse used by women to further reinforce their decision not to attend screening:

*I think people that weren’t going to come already have preconceived ideas and already have their excuses…and they will just use this [BC-Predict] for reinforcement if they wanted (Breast Screening Office Manager 2 – site C)*

To mitigate this, it was recommended that an option to speak to a HCP could help ease concerns and enable women to make informed choices about screening and risk; however, participants did not identify who would be best to deal with this:

*…there are sometimes things which patients won’t get involved with because of initial anxiety, not because they don’t believe in it […] whereas, if they have the opportunity to discuss it, that will make it less intimidating to be part of (Consultant Radiologist 1 – site A)*

#### Further marginalising underserved groups

Participants identified many barriers to breast screening engagement, with the most commonly cited being low socio-economic status, ethnicity, and learning difficulties. It was suggested that additional engagement required for risk stratification may deter these groups further:

*I think the level of involvement that would be required from a patient to get on board with this at the outset, may potentially further disengage a certain cohort of vulnerable patients… we need to make sure we don’t alienate them or…make it easier to disengage’ (Consultant Radiologist 1 – site A)*

Some participants suggested that women from the populations who disengaged from screening were likely to be most at risk of developing breast cancer and would benefit significantly from risk stratification, thus making accessibility imperative:

*The women that don't do the Predict thing [risk assessment], is it because they don't understand? So are they actually, could they be the group that are more at risk, because of their lifestyle… (Radiographer 5 – site B)*

#### Accessibility of additional screening

Participants stated that current guidelines for the management of women who are identified at high-risk of breast cancer are vague and inconsistent, *‘And they still haven’t sorted out high-risk protocols properly, have they?’ (Breast Screening Office Manager 2 –site C).* This has led to guidelines being subjectively interpreted to suit specific clinics:

*we’ve chosen to interpret the guidelines if you are moderate or high-risk then you will have annual mammograms until the age of 59 because it was easier to do that. It’s just that it’s very woolly isn’t it (Clinical Nurse Specialist 1 – site B).*

This incongruence between guidelines and the varying implementation at different screening sites was a concern. It was suggested that women identified as high-risk would receive different levels of care based on their location, which could have emotional, financial and capacity implications.

*…you’ll worry some people who can afford to go and do that [have extra screening privately]. And other people just here’s the anxiety and no funding (Consultant Radiologist 1 – site A)*

This highlights the need for clarity in the guidelines for classification, care and management of women, and to give specific instruction to breast screening units and family history risk and prevention clinics on how to care for women identified at increased risk.

#### Ambiguity in chemoprevention responsibility

A major concern at sites A & C was the lack of clarity as to who is responsible for prescribing preventative medication. Nursing staff across all sites were not licensed to prescribe preventative medication and as such there was a firm belief that this responsibility should lie with GPs, *‘I suppose the chemoprevention as well ties in with primary care…’ (Clinical Nurse Specialist 6 – site C).* However, the two GPs did express resistance towards prescribing preventative medication due to lack of time, *‘We’ve got enough to do, no, we haven’t got…we can’t’ (GP 1 – site A),* as well as insufficient knowledge:

*I think we have very limited knowledge of who has Tamoxifen and Raloxifene, so we're not going to be able to answer that question (GP 2 – site B).*

It was considered that women who opt for chemoprevention could be met with uncertainty regarding accessing risk management, while family history risk and prevention clinics operate without specific guidelines as to who will provide chemoprevention.

## Discussion

This study highlights the key drivers in healthcare staff attitudes to risk stratified breast screening as concerns surrounding capacity and service implementation at all points along the breast screening pathway. Participants saw value in providing women with personalised risk and highlighted the need for skilled communication of risks and service developments to ensure that stratification could be implemented in financially and time constrained settings without impacting negatively on women.

The central theme of pre-existing capacity and resource constraints drove concerns about providing breast cancer risk at screening and the additional complexity this adds to the service structure. Apprehension regarding NHS capacity in terms of clinical space and workforce is echoed in a recent report into the health of UK Cancer Services [12] which identified the financial environment to be an immediate challenge for cancer services with increasing numbers of referrals. More referrals to family history clinics as an outcome of identifying women at increased risk, the resulting risk management and associated costs were considered additional stressors. Although the concept of risk provision was favourable, this could not be separated from the identified capacity issues, thus resource constraints dominated and drove the discussion around risk stratified screening more so than concerns about the specifics of risk stratification.

Participants did not reach consensus in their views about providing women with notification of high-risk via letter. This was anticipated to be a potential source of service pressure, where women would rely on more frequent contact with HCPs. Yet, it was acknowledged that providing women with face-to-face risk consultation is equally problematic for capacity and is likely to be more costly. In contrast, primary care participants identified that high-risk notification for other diseases is provided via letter, so did not view this approach as problematic. These debates have appeared in many previous studies with HCPs, and indicate a tension between service capacity and the desire to avoid harms of screening [13], as well as concerns over potential harms through reducing screening to low risk women [14]. It is unclear whether the differing attitudes in our sample to providing risk via letter stems from over-caution, or whether there is a justifiable concern to be mindful when discussing cancer to minimise potential distress.

Fatalistic illness perceptions of cancer have been shown to impact on screening attendance and engagement in protective behaviours [15, 16]. However, provision of risk information in written format, particularly when followed by telephone consultation has been shown to increase screening uptake and improvement in psychological outcomes in both breast cancer and colorectal cancer screening [17, 18], drawing attention to the need for carefully constructed written risk information that accounts for illness perceptions and providing the opportunity for a follow-up call. The content and format of risk information was discussed by our participants, with a consensus of opinion that careful wording would guard against increased anxiety, thus having a knock-on effect on workload of breast screening staff. Indeed the provision of personalised risk information promotes informed decision-making [19] and increases knowledge without decreasing emotional wellbeing [20, 21]. However, it is yet to be determined how risk provision impacts on help-seeking behaviour in women attending routine mammography.

Participants warned against accentuating inequity by overburdening women with a complex risk estimation process that is heavily reliant on written information, particularly if women view completion of a risk questionnaire as necessary to access breast screening. It should be noted that some participants identified disadvantaged groups, such as those of lower socio-economic status with lower health literacy as being more at risk of cancer, which is not fully in accord with current evidence. It is certainly the case that health literacy varies widely in the UK [22] and poor health literacy has been consistently associated with lower screening attendance and healthcare usage [23]. Further, women with disabilities [24] and those with lower household wealth [25] are less likely to attend breast screening. However, only some of these groups are at greater risk of breast cancer, e.g. higher SES women may have higher risk [26]. Despite this, there is a need to be cautious when introducing risk stratification into the NHS-BSP as it may impact further on screening uptake for disadvantaged groups, especially those with low health literacy. In addition, HCPs were concerned about the potential impact on anxiety of providing women with their risk of developing breast cancer at screening. Available literature which has examined the harms and benefits of receiving risk on psychological variables [27], has shown that there were no major harms in providing breast cancer risk to women [28, 29].

The study is somewhat limited by risk stratification being hypothetical to the participants at this stage Previous qualitative studies with healthcare professionals in other countries have investigated this topic, and also identified concerns about healthcare system capacity [30], how best to communicate risk information [31] and exacerbating inequities [32]. It should be noted however that the participants in the present study were aware that they would be implementing the care pathways discussed in the near future, which clearly focussed minds on how to make the pathways work.

Recruitment of Primary Care participants proved challenging, contributing to the high rate of ‘no responses’ to the study invitation. Future research outside of the remit of this programme should focus on how risk estimation could be implemented into routine screening without disengaging minority and underserved groups, whilst promoting uptake to screening. Research should continue to investigate ways in which to communicate breast cancer risk to women in clear, effective and sensitive ways to minimise distress, anxiety and worry.

## Conclusion

The results of this study highlight the key drivers in frontline breast screening healthcare professional attitudes to providing breast cancer risk at routine mammography as concerns surrounding capacity and service implementation at all points along the breast screening pathway, including Primary Care. Participants saw value in providing women with personalised risk and highlighted the need for skilled communication of risks and service developments to ensure that services could be implemented in financially and time constrained settings without impacting negatively on the women receiving risk information. Implementing risk stratification into the NHS-BSP or other healthcare systems needs to consider as priority improving capacity and demands on healthcare professional time, shortages of trained staff to share and reduce workload burden, effective and appropriate communication strategies for breast cancer risk and pathways of care.

## Supplementary Information

**Additional file 1.** COREQ checklist.

**Additional file 2.** Coding tree.

## Data Availability

The dataset analysed is not publicly available as the data may contain information that would compromise participant consent, i.e. allow individuals to be identified. Bone fide researchers should please contact the corresponding author to discuss access to the dataset.

## Abbreviations

BC-Predict: Breast Cancer Predict
NHS: National Health Service
NHS-BSP: National Health Service Breast Screening Programme
