# Supplementary material for: The feasibility of implementing risk stratification into a national breast cancer screening programme: a focus group study investigating the perspectives of healthcare personnel responsible for delivery
Source: BMC Womens Health. 2022 May 2;22:142. doi: 10.1186/s12905-022-01730-0 (PMC9063090; doi:10.1186/s12905-022-01730-0)
Supplement: Supplementary file 1 [file 12905_2022_1730_MOESM2_ESM.docx]

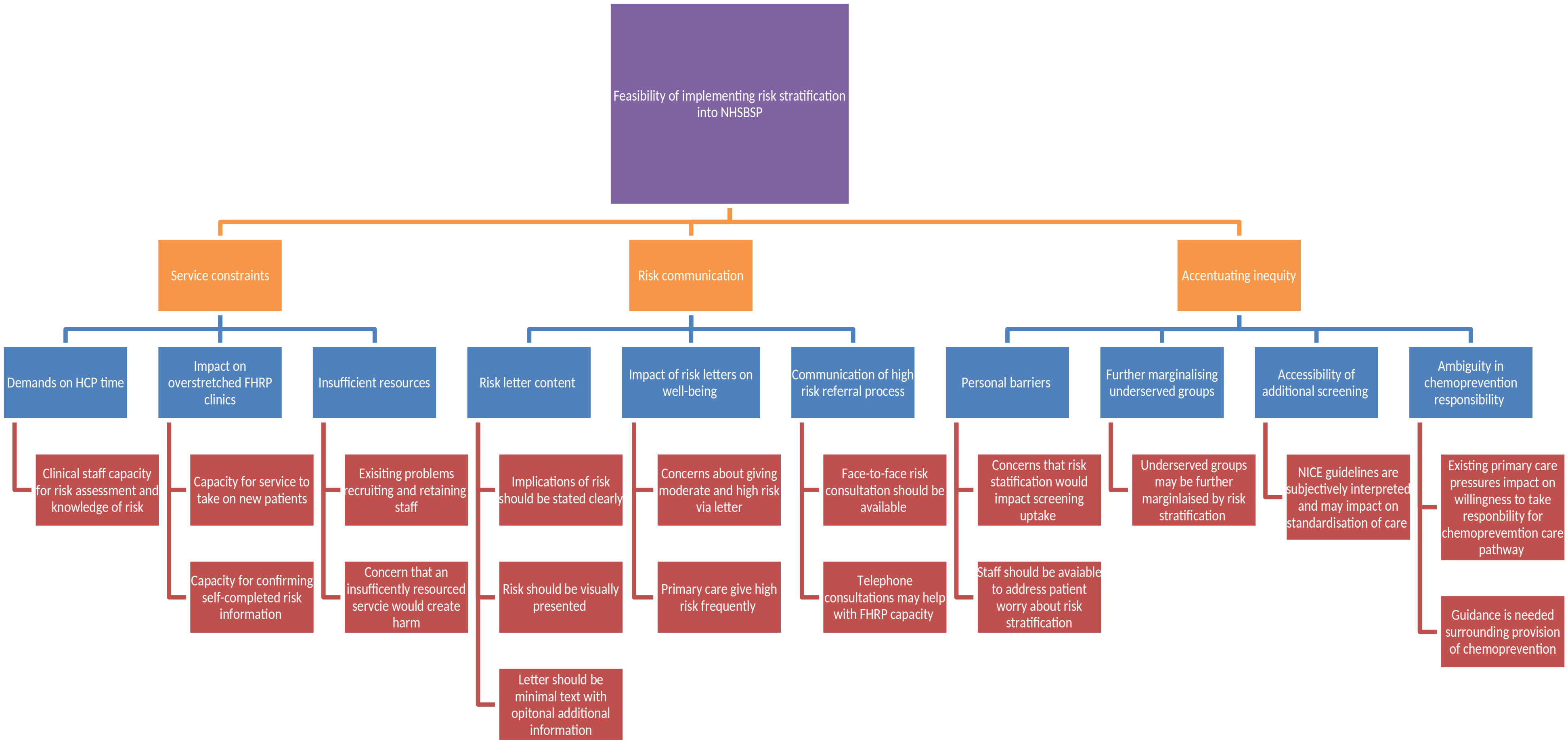

Feasibility of implementing risk stratification into NHSBSP
Service constraints
Risk communication
Accentuating inequity
Demands on HCP time
Impact on overstretched FHRP clinics
Insufficient resources
Risk letter content
Impact of risk letters on well-being
Communication of high risk referral process
Personal barriers
Further marginalising underserved groups
Accessibility of additional screening
Ambiguity in chemoprevention responsibility
Clinical staff capacity for risk assessment and knowledge of risk
Capacity for service to take on new patients
Capacity for confirming self-completed risk information
Exisiting problems recruiting and retaining staff
Concern that an insufficently resourced servcie would create harm
Implications of risk should be stated clearly
Risk should be visually presented
Letter should be minimal text with opitonal additional information
Concerns about giving moderate and high risk via letter
Primary care give high risk frequently
Face-to-face risk consultation should be available
Telephone consultations may help with FHRP capacity
Concerns that risk statification would impact screening uptake
Staff should be avaiable to address patient worry about risk stratification
Underserved groups may be further marginlaised by risk stratification
NICE guidelines are subjectively interpreted and may impact on standardisation of care
Existing primary care pressures impact on willingness to take responbility for chemoprevention care pathway
Guidance is needed surrounding provision of chemoprevention
